# Improvements in Glycemic, Micronutrient, and Mineral Indices in Arab Adults with Pre-Diabetes Post-Lifestyle Modification Program

**DOI:** 10.3390/nu11112775

**Published:** 2019-11-15

**Authors:** Hanan Alfawaz, Alsoodeeri Fahadah Naeef, Kaiser Wani, Malak Nawaz Khan Khattak, Shaun Sabico, Abdullah M. Alnaami, Nasser M. Al-Daghri

**Affiliations:** 1 Department of Food Science and Nutrition, College of Food Science and Agriculture King Saud University, Riyadh 12372, Saudi Arabia; halfawaz@ksu.edu.sa (H.A.); fahdah-n@hotmail.com (A.F.N.); 2 Biochemistry Department, College of Science, King Saud University, Riyadh 11451, Saudi Arabia; wani.kaiser@gmail.com (K.W.); malaknawaz@yahoo.com (M.N.K.K.); eaglescout01@yahoo.com (S.S.); aalnaami@yahoo.com (A.M.A.)

**Keywords:** pre-diabetes, lifestyle modification, nutrition guidance, minerals, micronutrients, recommended dietary intakes

## Abstract

The present study aimed to investigate the changes in dietary patterns of adult Saudis with prediabetes who underwent a six-month lifestyle modification program. A total of 160 Saudis with prediabetes (baseline fasting glucose 5.6–6.9 mmol/L), aged 20–60 years, were enrolled in one of the two arms: A one-time general advice about lifestyle modification (GA group) at orientation or a well-structured and monitored nutrition and lifestyle counseling for six months (guidance group). Fasting blood samples and a dietary recall for daily intakes of macro/micronutrients using a validated computerized food database “ESHA—the Food Processor Nutrition Analysis program” were collected pre- and post-intervention. Compliance to reference daily intake (RDI) was also calculated at both time points. At baseline, overall, severe deficiencies in the majority of micronutrient intakes were observed. Post intervention, clinically significant improvements in the glycemic indices (fasting glucose and insulin resistance) were seen over time in the guidance group. Also, significant improvements in dietary habits and physical activity levels were more apparent in the guidance group than the GA group, particularly in the daily intakes of total carbohydrate (46.9% compliance post vs. 20.3% at baseline); dietary fiber (21.9% vs. 3.1%); and some micronutrients like vitamin B6 (21.3% vs. 6.7%), vitamin B12 (45.3% vs. 28%), vitamin C (21.9% vs. 7.8%), riboflavin (40% vs. 10.7%), niacin (41.3% vs. 14.7%), magnesium (18.8% vs. 4.7%), iron (54.7% vs. 34.4%), and copper (37.3% vs. 13.3%). The study highlights the effects of a six-month lifestyle modification program in improving dietary micronutrient intakes of Saudis with prediabetes. Since micronutrient intake was observed to be low, fortification of these micronutrients in the Saudi diet is recommended.

## 1. Introduction

Diabetes mellitus (DM) is one of the most challenging health problems of the 21st century, with 1 in 11 adults having diabetes worldwide and an estimated 425 million diabetics in the age range of 18–99 [1]. It is predicted to rise to 642 million by 2040, posing a big impact on the health care cost of the country [2]. A significant opportunity to reduce such impact lies in focusing on people with intermediate hyperglycemia (prediabetes). The American Diabetes Association (ADA) defines prediabetes as having either impaired fasting glucose (IFG), defined as fasting plasma glucose (FPG) between 5.6–6.9mmol/L, or impaired glucose tolerance (IGT), defined as post-prandial, 2 h plasma glucose between 7.8 and 11.0 mmol/L on oral glucose tolerance test (OGTT) [3]. Around 5%–10% of people with prediabetes eventually progress to type 2 DM (T2DM) [4]. Saudi Arabia is not spared in the widespread increase in the prevalence of T2DM, with the leading cause being the rising obesity rate, genetic predisposition, and an aging population [5]. In fact, a recent report from the International Diabetes Federation (IDF) mentioned that Saudi Arabia is one of the top 10 countries with the highest prevalence of T2DM among adults [6]. Risk factors for T2DM, apart from age and family history, includes obesity, physical inactivity, and over nutrition [7].

Fortunately, lifestyle modification interventions such as diabetes prevention programs led by the National Institute of Diabetes [8] revealed that modifications like a balanced diet and increased physical activity are the gold standards when it comes to reducing the incidence of T2DM and even in its management, as these programs help to improve blood glucose control, body weight, and insulin resistance. Increased public awareness of the disease and its risk factors, emphasis on timely screening, and patient education on lifestyle modification (diet and physical activity) improves preventive service delivery [9]. However, recent meta-analyses [10,11], time and again, conclude that more interventional studies identifying different components associated with increased effectiveness of these lifestyle modification programs are required to help to devise them in a better and more economical manner. Components like education on dietary nutrients and mineral indices, usefulness of these indices in reducing the glycemic load, and an overall education on increased physical activity levels and its impact on weight reduction would, in the long run, help to address the issue of rising prevalence of impaired glucose tolerance in populations. In that context, a study on the changes in dietary patterns post-lifestyle modifications may provide insight into how to tailor these programs in a better and more useful manner.

Dietary patterns, assessing comprehensive information about intake of dietary nutrient and mineral indices, are a new focus in nutritional epidemiology, and some groups, including us, reported on the relationship between different dietary patterns and DM [12,13,14]. However, the data on the effects of lifestyle modification education on the dietary patterns of people with prediabetes are limited. Our earlier lifestyle modification studies on pre-diabetic Saudi adults also lacked the dietary recall data and, hence, the changes in dietary patterns could not be assessed [15,16]. To address this, in this study, we aimed to investigate the effects of nutritional education and lifestyle modification program on the dietary patterns of people with prediabetes in Saudi Arabia. We also aimed to characterize the beneficial effects of these programs on glycemic indices vis a vis changes in dietary patterns.

## 2. Materials and Methods

### 2.1. Study Design and Participants

**Figure 1:**
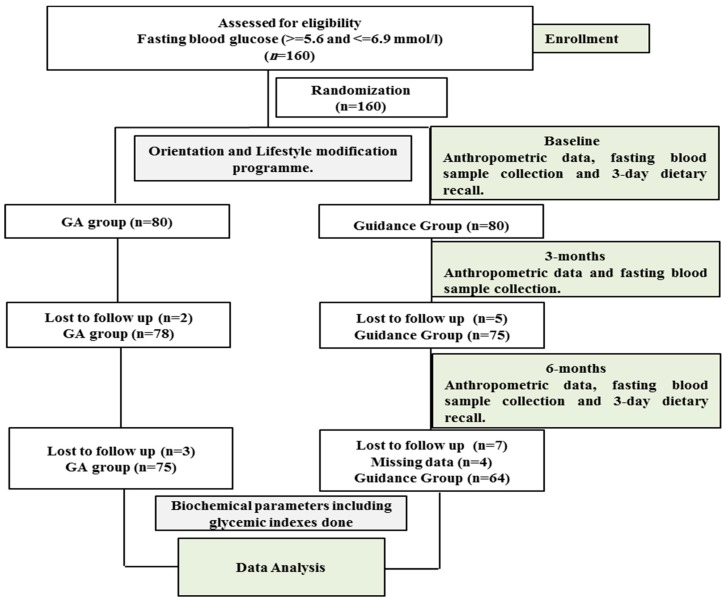
Flow chart detailing the participation of participants and their allocation to treatment groups.

This is a multi-center, 6-month interventional study that was carried out in Al-Quds Health Care Center and selected schools in Riyadh city from April 2016 to March 2018. A total of 160 Saudis with known prediabetes aged 20–60 years were enrolled to participate. Signed consent forms were obtained prior to inclusion and ethical approval (Reference# 8/25/220355) was obtained from the Ethics Committee of the College of Science, King Saud University, Riyadh, Saudi Arabia. Inclusion criteria included participants with a fasting glucose level of 5.6–6.9 mmol/L. Exclusion criteria included non-Saudis; participants who were on anti-hyperglycemic drugs; pregnant and breastfeeding women; children and those who were unable to answer the questionnaire independently; as well as those with known cardiovascular diseases, liver and kidney diseases, diabetes, and/or malignancies. Participants were assigned to either of the two groups by allocating them a computer generated random serial numbers. Apart from the anthropometric data and fasting blood samples collected from each participant at recruitment, after 3 months and at end of the study (6 months) dietary recall data were also obtained from each participant at recruitment and end of the study. A flow chart of the study program is given in Figure 1.

### 2.2. Intervention

The first group (GA) received general information about risk factors of prediabetes and DM. The idea of the prevention of type 2 diabetes by introducing lifestyle changes was briefed to them at orientation. The other group (guidance) received well-structured nutrition and lifestyle counseling about prediabetes, DM, nutrition, weight management, and physical activity. The lectures were performed during the regular visits of the participants in their respective centers. Two such visits were done by each participant in the intervention group, one at baseline and the other after three months in the program. In the first visit, participants received booklets and brochures about prediabetes. In addition, they attended workshops about diabetes. The intervention group was given three lectures per visit. These lectures included the definition of prediabetes, risk factors, complications, management plan, and measures for preventing further complications. They were also educated about self-care, food items that were low in carbohydrates and high in fiber, as well as substitutes such as replacing animal fats with vegetable oils available in the market. Knowledge on increased physical activity was also provided. This included 20–30 min of various activities such as running/jogging at least three times a week. They also received posters, leaflets, video films as audiovisual aids, as well as pamphlets to remind them of the education session with a focus on nutrition and physical activity.

### 2.3. Questionnaire Design and Output

The questionnaire was designed to contain three parts: The first part included the demographics (age, sex, marital status, education level, occupation, etc.) and anthropometrics like weight, height, systolic and diastolic blood pressure, etc., which was measured by standard methods like a standardized digital scale and measuring tape, etc. BMI was calculated by the standard formula of weight in Kg/(height in m)^2^. A BMI of >25.0 and <30.0 was considered as overweight, and 30.0 and higher was considered as obese. The second part included information on dietary preferences like full/low fat dietary products, red/white meat, white/whole bread, etc., and was recorded as frequency; it also contained questions on physical activity recorded as frequency of at least 20 min of moderate/vigorous physical activity per week. The last part was specific to 24 h dietary recall in which a structured interview captured the detailed information of all foods and beverages consumed by the participant within 24 h during the 3 days of the week including one day at the weekend. The participants were supplied with some standard dishes, cups, and spoons; food models; and pictures for accuracy of input. These measurements were done at baseline and at end of the study. The dietary recall methodology included face-to-face interview of the study participants by a trained dietician to assess the food consumption, as done earlier by the research team [17].

The food preference and physical activity questionnaire data recorded as daily/once a week/twice a week/never were transformed to a scale of 0–7 with 0 and 7 representing none and all days in a week, respectively. The food frequency questionnaire recorded as dietary recall was used to assess total calories, macronutrient, and micronutrient intake using a validated computerized food database “ESHA—the Food Processor Nutrition Analysis program” [18]. Reference daily intake (RDI) was used to calculate the percentages of participants following these recommendations for each macro/micro nutrient at recruitment and post intervention. Recommended dietary allowance (RDA) was used for nutrients where the defined tolerable upper limit (UL) is absent. RDI energy was calculated from estimated energy requirement [19] by replacing the actual body weight (kg) by ideal body weight [20]. RDI for each macro/micro nutrient was estimated by using recommendations by institute of medicine of the national academics [21].

### 2.4. Determination of Biochemical Parameters

Fasting venous blood was collected at baseline, and after 3 and 6 months from each participant; centrifuged to obtain serum; and transported to the Chair for Biomarkers of Chronic Diseases (CBCD), King Saud University (KSU) for further analysis. Serum lipid profile and glucose was determined using automated biochemistry analyzer (Konelab 20, Thermo-Fischer Scientific, Helsinki, Finland). HbA1c was measured in DCA vantage analyzer (Siemens, Munich, Germany). Fasting insulin was quantified by Luminex multiplex (Luminexcorp, Austin, TX, USA) using fluorescent microbead technology. The intra, inter assay variation for HbA1c test and insulin was 2.6%, 4%, and 4.2%, 5.4%, respectively. Homeostatic model assessment for insulin resistance (HOMA-IR) was calculated as [fasting insulin (mU/L) × fasting glucose (mmol/L)/22.5)] [22].

### 2.5. Statistical Analysis

The data were analyzed using SPSS version 23. Data were presented as mean ± SD for continuous normal variables; medians (25th percentile, 75th percentile) for continuous non-normal variables; and N (%) for categorical variables. Baseline differences between groups was done using independent Student T-test and Mann–Whitney U-test for Gaussian and non-Gaussian variables, respectively, and χ² test for categorical variables. Differences within groups were calculated using repeated measures t-test and Wilcoxon signed-rank test for Gaussian and non-Gaussian variables, respectively. Dietary preferences and physical activity (frequency/week) and dietary recall data (as macro/micronutrient intake/day) were presented as median (25th percentile, 75th percentile) and the difference estimated by Wilcoxon signed-rank test. Analysis of covariance (ANCOVA) was done to determine differences between groups. Lastly, RDI or RDA was used to calculate the percentages of participants following these recommendations for each macro/micro nutrient and the data were given in percentages; the difference within groups at end of study was compared with baseline by McNemar chi-square test. *p*-value < 0.05 is considered significant.

## 3. Results

### 3.1. Baseline Characteristics of Study Groups

Table 1 shows the baseline anthropometric and clinical characteristics of study participants. A total of 139 prediabetes patients (67 males and 72 females) (guidance group: N = 64 (26 males; 38 females) and GA group: N = 75 (41 males; 34 females)) were included. Most of the participants (84% in GA and 93.7% in guidance) were in the age group of 30–49. Furthermore, 93.3% participants in the GA group and 90.6% in the guidance group were either overweight or obese. At baseline, the general and clinical characteristics of the two study groups were comparable especially in distribution of age group; sex; overweight and obese proportion; fasting glucose, HbA1c, insulin levels, and HOMA-IR. However, the two groups at baseline had slight differences in their mean BMI (*p* = 0.04). Some of the other anthropometric indexes and lipid profile results at baseline are shown in Supplementary Table S1.

### 3.2. BMI, Glycemic Profile, and Physical Activity Changes of Study Groups Overtime

Table 2 shows the changes in BMI, glycemic profile, and physical activity levels overtime. Baseline and six-month data were used to construct this table. In the guidance group, BMI reduced significantly over time (*p* < 0.01) while in GA group, BMI increased slightly over time (*p* < 0.05). Between-group comparison showed no statistical difference in BMI. With regards to glycemic indices, fasting glucose and HbA1c significantly reduced overtime in the guidance group but not in the GA group. Insulin was reduced significantly in both groups over time (*p* < 0.05 and < 0.01 in GA and guidance groups, respectively). HOMA-IR was reduced significantly only in the guidance group [median (Q1, Q3) of 4.51(4.2, 5.2) at baseline to 4.09 (3.4, 4.7) at end of study, *p* < 0.01]. Between-group comparison showed clinically significant differences in favor of the guidance group in terms of fasting glucose (*p* = 0.005), HbA1c (*p* = 0.005), and HOMA-IR (*p* = 0.034). Changes over time in other anthropometric and lipid indices are shown in Supplementary Table S2.

Table 2 also shows the changes in physical activity levels of the two study groups at baseline and end of study. In the guidance group, moderate physical activity (like walking at least 20 min/day or using stairs) increased from 2.3 times/week at baseline to 2.6 times/week (*p* = 0.03) after six months and vigorous physical activity (like regular exercise or swimming or sports) increased from 0.40 times to 1.40 times/week (*p* < 0.01). The dietary preferences and physical activity levels remained more or less unchanged in the GA group. Between-group comparison showed a clinically significant difference in vigorous physical activity in favor of the guidance group. Supplementary Table S3 shows the changes in dietary preference of the study participants calculated from the food questionnaire.

### 3.3. Changes in Macro/Micronutrient Intakes Overtime

Table 3 shows the dietary intake of macro and micronutrients at different time points. The average intake of macronutrients over time comparable in both groups except carbohydrates where the average intake decreased significantly over time by 24.2 g/day (*p* = 0.05) in the guidance group. The average intake of dietary fiber increased significantly over time by 5.4 g/day (*p* < 0.01) in the GA group and by 4.8 g/day (*p* < 0.01) in the guidance group. No significant changes in protein and fat consumption were observed in both groups, although between-group comparison showed a clinically significant decrease in saturated fat intake in favor of the guidance group (*p* = 0.04). The average intake of all the micronutrients increased significantly in the guidance group, except zinc. The average consumption of dietary vitamin A overtime increased by 211.04 μg RAE/d (*p* = 0.001) in the guidance group. Dietary vitamin C and E consumption also increased by 9.84 mg/day (*p* < 0.001) and 2.55 mg/day (*p* < 0.001), respectively, as well as thiamine, riboflavin, niacin, B6, and folate in this group. Furthermore, the average dietary intake of calcium, phosphorus, magnesium, iron, copper, sodium, and potassium increased significantly over time in the guidance group. In the GA group, significant increase in consumption of vitamin B6, folate, vitamin E, and essential minerals like phosphorus, magnesium, copper, and potassium was observed. Lastly, between-group comparisons revealed a clinically significant increase in riboflavin (*p* = 0.01), niacin (*p* < 0.001), vitamin B6 (*p* < 0.001), vitamin B12 (*p* = 0.041), vitamin E (*p* = 0.003), phosphorus (*p* < 0.001), magnesium (*p* < 0.001), iron (*p* = 0.01), copper (*p* = 0.03), sodium (*p* = 0.01), and potassium (*p* = 0.01), all in favor of the guidance group.

### 3.4. Changes in Dietary Compliance of the Study Groups Overtime

Table 4 shows the changes in the percentages of the study participants following the criteria of recommended dietary allowances of macro and micronutrients as given by the National Academy of Medicine. A general improvement was seen in the guidance group as compared to the GA group. Specifically, 20.3% of participants in the guidance group at baseline followed the recommendation of limiting carbohydrate intake of 35% of RDI energy, which increased to 46.9% (*p* = 0.003) at the end of the study. Similarly, participants following recommendation of taking at least 25 g/day of dietary fibers increased from 3.1% to 21.9% (*p* = 0.002) in the guidance group and from 2.7% to 21.3% (*p* = 0.001) in the GA group. As far as micronutrients are concerned, a general increase in percentage of participants following these recommendations was seen in the guidance group with statistically significant increase in vitamins like riboflavin, niacin, B6, B12, and vitamin C and essential minerals like magnesium, iron, and copper. In GA group, the participants who followed dietary recommendations set for phosphorus and iron increased significantly from baseline to end of study while for sodium, it decreased significantly overtime. Also, the participants who followed dietary recommendation for vitamins like vitamin A, thiamine, folate, and vitamin E and essential minerals like calcium, magnesium, potassium, and zinc was very low and did not improve much over time in both intervention groups.

## 4. Discussion

In this study, we investigated the changes in the dietary patterns of Saudi adults with pre-diabetes following a six-month lifestyle modification program. Changes in glycemic indices in relation to changes in dietary patterns were also investigated. To the best of our knowledge, this is the first such study done among Arab populations with prediabetes. The study demonstrated a significant reduction in total carbohydrates consumption and a significant increase dietary fiber intake in favor of the guidance group post intervention. Furthermore, a general significant increase in the overall micronutrient intake was observed in the guidance group post intervention. Significant improvements in vigorous physical activity was observed in this group as well. These favorable changes in the guidance group may explain the significant improvements in glycemic indices post intervention.

A growing interest is evident in the examination of dietary/food patterns among populations at risk because of its usefulness in the prevention and treatment of chronic diseases, including DM [23]. Even if food patterns vary widely across countries, they can nevertheless be quantified as energy intake, carbohydrates, fat, protein, etc., [24,25] to monitor the changes post intervention. Despite the American Diabetes Association’s (ADA) recommendation that macronutrient composition and meal plans should be based on individual preferences and needs, many clinicians continue to prescribe a low-fat meal plan for DM management. The “Diabetes Plate” approach contains three to five carbohydrate choices per meal, one to three starch choices, one milk serving, and one piece of fruit [26,27]. Milk and yogurt consumption also have a role in reversing insulin resistance in people with prediabetes. In one study [28], the effects of dairy consumption on lipids, glucose, and insulin among 23 males and females aged between 18–75 years old were examined. It suggested that under free-living conditions, consumption of four servings of low-fat dairy milk and yogurt products for six months may improve insulin resistance without inversely impacting bodyweight or lipid status. In another recent study [29], different dietary patterns among 8693 participants found a direct association between animal protein intake and presence of DM. These findings support the findings of this study where a significant improvement in glycemic indices in the guidance group was parallel to the significant reduction in consumption of full fat dietary products; a significant increase in consumption of low-fat dietary products; and a significant increase and decrease in intake of whole-meal bread and white bread, respectively (Table S3).

Low physical activity and sedentary lifestyle, apart from the unhealthy dietary habits, is one of the main risk factors for DM [30]. Due to the enormous economic growth during the last few decades, the sedentary lifestyle has undoubtedly become increasingly prevalent in all sections of Saudi society [31]. Physical activity is important, especially in preventing diabetes, as it enhances insulin sensitivity and improves glycemic control [32]. Regular physical activity also enhances insulin action in muscle and liver leading to decrease blood glucose [33]. The increased frequency of physical activity per week in the guidance group may be attributed to a systemic knowledge sharing on the health benefits of physical activity in the guidance arm of this study. However, the mean physical activity level of the participants at the end of this six-month intervention program fell short of the standards recommended by WHO [34]. More aggressive efforts are needed to impart the knowledge on the health benefits of physical activity, starting at the level of schools and extending it to community settings and workplaces. Healthcare providers in Saudi Arabia have an important role in promoting physical activity and so do the national policy makers in establishing and encouraging activity living in all spheres of day to day life activities.

The three macronutrients (carbohydrates, fat, and protein) are the main sources of energy derived from food [35]. In this study, a significant decrease in per day intake of carbohydrates was observed in the guidance group (Table 3) and also the percentage of participants following the dietary recommendations for carbohydrates increased significantly post intervention from 20.3 to 46.9 (*p* = 0.003) (Table 4). Carbohydrates, the primary macronutrient of concern in glycemic management, has a direct effect on postprandial glucose levels in patients with impaired glucose regulation; however, the quality of carbohydrates ingested may also be of primary importance. The ability of nutrients to raise glucose levels seems to be directly linked with its gastrointestinal transit and velocity of absorption [36]. As such, the dietary fibers, which are the indigestible components of complex carbohydrates, are unanimously suggested to be protective against risk of diabetes [37]. In this study, the authors observed a significant increase in intake of dietary fibers in the guidance group and also the participants following recommendations (at least 25 g/day) in this group increased from 3.1% at baseline to 21.9% at end of study (*p* = 0.002) (Table 4). Increased intake of dietary fibers appears to improve insulin action by an increased secretion of glucose-dependent insulinotropic polypeptide (GIP), an incretin hormone that inhibits glucagon secretion [38]. Regarding fat consumption, even though there is no conclusive evidence that links the reduction of fat consumption with incidence of type 2 diabetes [39], reducing overall calorie intake including replacement of saturated fats with polyunsaturated fatty acids may be useful for weight management in lifestyle modification programs [40]. Our data showed that in between-group comparison, a significant reduction in the consumption of saturated fats was observed in favor of the guidance group (*p* = 0.04) (Table 3).

The current study highlights the importance of lifestyle modification programs in educating people with prediabetes to acquire daily vitamin and nutrient requirements from natural food sources. Nutrition therapy has been accepted as a cornerstone in the management of diabetes, yet uncertainty still exists especially in the role of micronutrients related to diabetes and its complications [41]. The authors’ acknowledge the vast and diverse nature of this topic, which is beyond the scope of this study. However, it must be stated that some epidemiological studies have documented the association of poor intake of some of the micronutrients like magnesium; potassium; zinc; and vitamin B6,B12, C, etc., and deficient glycemic control, and patients with impaired glucose regulation are found to be susceptible to multiple micronutrient deficiencies [42]. Nevertheless, the potent antioxidant properties of some of these micronutrients like riboflavin, niacin, vitamin E, etc., is widely accepted and their ingestion at recommended levels could delay or reverse the oxidative damages of a hyperglycemic state [43].

The results of this study also highlight the low intakes of some of these micronutrients in a normal Saudi diet. Average dietary intakes of micronutrients such as vitamin A, vitamin E, thiamine, folate, calcium, and potassium were much lower than the recommended intakes in both time points. Such low dietary compliance may be because many of these nutrients are less available in natural foods and this fortification has not been taken into consideration by “ESHA”. Regardless, the results of the study reflect the need to introduce foods rich in these micronutrients to the Saudi dietary habits and also a need to have a good food fortification policy, as implemented successfully in many Western countries. Lifestyle intervention programs on people with high risk for diabetes should be designed to maximize the compliance for recommended dietary intakes of micronutrients. Additionally, micronutrient supplements for some selected group of individuals like the elderly and strict vegetarians as recommended by the ADA could be incorporated in the intervention program.

The authors acknowledge some limitations in the current study. First, this intervention program was designed for a group of individuals with impaired glucose regulation from an ethnically homogenous cohort, the results may not apply to other populations, e.g., people with established DM. Second, the dietary recall data analyzed in this study are subject to recall and social desirability biases. Third, the diet history questionnaires used in this study may not have included all types of foods/beverages consumed by the participants and in such cases, best possible estimates would have been selected. Lastly, the food calorie calculator used in this study to estimate the macro/micronutrient estimates may not have all the foods/beverages consumed listed in its database. Despite these limitations, this study has merits in being the first to investigate the acute changes in dietary patterns in Saudi adults with prediabetes following a six-month lifestyle modification program.

## 5. Conclusions

In summary, this study showed the beneficial effects of a six-month lifestyle modification program on improving macro/micronutrient intakes and physical activity levels among Saudi adults with prediabetes. The study also sheds light on the poor dietary intake of essential micronutrients in the Saudi diet and, as such, dietary fortification is recommended.

## Figures and Tables

**Table 1:** General, anthropometric, and biochemical characteristics of study groups at baseline.

Parameters	GA (N = 75)	Guidance (N = 64)	*p*-Value
**Age group (year)**			
20–29	7 (9.5)	2 (3.2)	
30–39	34 (44.6)	26 (41.3)	
40–49	29 (39.2)	34 (52.4)	NS
50–60	5 (6.8)	2 (3.2)	
**Marital Status**			NS
Married	64 (85.1)	56 (87.5)	
Not married	11 (14.9)	8 (12.5)	
**Education**			NS
Elementary	3 (4.1)	2 (3.3)	
Secondary	2 (2.7)	2 (3.3)	
Undergraduate	16 (21.9)	17 (26.2)	
Graduate	47 (61.6)	40 (53.9)	
Post graduate	7 (9.6)	3 (3.5)	
**Sex**			NS
Male	41 (54.7)	26 (40.6)	
Female	34 (45.3)	38 (59.4)	
Overweight	37 (49.3)	24 (37.5)	NS
Obese	33 (44.0)	34 (53.1)	
Age (years)	43.3 ± 6.7	43.4 ± 5.6	NS
BMI (kg/m^2^)	30.4 ± 4.3	32.3 ± 6.2	0.04
Fasting Glucose (mmol/L)	6.1 ± 0.8	6.0 ± 0.7	NS
HbA1C	5.6 ± 0.8	5.6 ± 0.6	NS
Insulin (μU/mL)	16.52 (15.7,18.1)	16.80(16.5,20.3)	0.06
HOMA-IR	4.36 (4.1,4.8)	4.51 (4.2,5.3)	0.36

**Note**: NS: *p*-value not significant in overall subgroups. HOMA-IR is homeostatic model assessment for insulin resistance. Data presented as mean ± SD for continuous normal variables; medians (25th percentile, 75th percentile) for continuous non-normal variables; and N (%) for categorical variables. The difference between groups at baseline was calculated by independent samples t-test and Mann–Whitney U-test for Gaussian and non-Gaussian variables, respectively, and χ² test for categorical variables. *p* < 0.05 is taken as significant.

**Table 2:** Body mass index (BMI), glycemic profile, and physical activity changes of groups over time.

Parameters	GA (N = 75)			Guidance (N = 64)			Interaction *p*-Value
	Baseline	6 months	*p*	Baseline	6 months	*p*	
BMI	30.42 ± 4.3	30.83 ± 4.3	0.02	32.34 ± 6.2	31.77 ± 6.7	0.002	0.53
Fasting glucose (mmol/L)	6.06 ± 0.8	5.87 ± 1.1	0.15	6.03 ± 0.7	5.70 ± 1.0	0.01	0.005
Hba1c (%)	5.62 ± 0.8	5.41 ± 1.1	0.07	5.61 ± 0.6	5.35 ± 1.0	0.03	0.005
Insulin (μU/mL)	16.52 (15.7,18.1)	15.98 (15.8,18.4)	0.02	16.80 (16.5,20.3)	16.05 (15.4,18.8)	0.003	0.06
HOMA-IR	4.36 (4.1, 4.8)	4.19 (3.8, 4.8)	0.07	4.51(4.2, 5.2)	4.09 (3.4, 4.7)	<0.01	0.034
Moderate Physical Exercise/week	2.00 (1.3, 3.0)	2.00 (1.5, 2.5)	0.89	2.3 (1.5, 3.0)	2.6 (1.3, 3.6)	0.029	0.07
Vigorous Physical Exercise/week	0.50 (0.3, 1.4)	0.60 (0.3, 1.3)	0.78	0.40 (0.2, 0.9)	1.40 (1.2, 1.6)	<0.01	0.017

**Note**: HOMA-IR is homeostatic model assessment for insulin resistance. Data presented as mean ± SD for continuous normal variables; medians (25th percentile, 75th percentile) for continuous non-normal variables. The difference within groups compared with baseline was calculated by repeated measures t-test and Wilcoxon signed-rank test for Gaussian and non-Gaussian variables, respectively. Interaction *p*-value represents the overall intervention effect between the groups. *p*-value < 0.05 is considered significant.

**Table 3:** Changes over time in nutrient intakes.

Parameters	GA (N = 75)			Guidance (N = 64)			Interaction *p*-Value
	Baseline	6 months	*p*	Baseline	6 months	*p*	
Energy (kcal/day)	2285.2 (2025, 2628)	2251.8 (1957, 2828)	0.97	2230.7 (1982, 2814)	2042.4 (1728, 2457)	0.11	0.20
Macronutrients							
Carbohydrate (g/day)	251.0 (198.7, 301.9)	243.1 (198.5, 326.3)	0.74	229.5 (197.2, 289.1)	205.3 (149.0, 263.8)	0.046	0.01
Total Fiber (g/day)	8.4 (6.2, 11.9)	13.4 (7.5, 17.5)	0.001	8.3 (5.0, 10.9)	13.1 (11.1, 16.6)	<0.001	0.45
Protein (g/day)	90.2 (70.2, 107.0)	91.4 (71.7, 106.8)	0.78	91.4 (71.7, 103.8)	82.5 (70.2, 95.2)	0.08	0.05
Total Fat (g/day)	72.8 (63.1, 92.9)	69.4 (60.3, 91.9)	0.23	70.0 (59.5, 84.3)	63.2 (57.0, 75.5)	0.12	0.06
SF (g/day)	25.4 (20.8, 33.6)	25.4 (19.8, 33.6)	0.61	25.5 (19.0, 32.1)	20.3 (14.1, 31.1)	0.09	0.04
MUFA (g/day)	25.1 (23.2, 31.7)	25.0 (20.6, 33.5)	0.53	25.0 (20.2, 29.8)	24.4 (18.7, 29.8)	0.49	0.12
PUFA (g/day)	17.4 (15.4, 23.6)	18.8 (15.4, 24.5)	0.21	19.8 (16.1, 24.2)	19.8 (16.1, 24)	0.94	0.14
**Micronutrients**							
Vit. A (μg RAE/d)	283.3 (253.2, 525.5)	370.4 (287.1, 504.1)	0.26	226.5 (139.9, 461.3)	437.5 (310.6, 547.1)	0.001	0.65
Thiamine (mg/day)	0.44 (0.4, 0.6)	0.49 (0.4, 0.7)	0.16	0.31 (0.2, 0.4)	0.66 (0.5, 0.8)	<0.001	0.81
Riboflavin (mg/day)	0.76 (0.6, 1)	0.80 (0.6, 1.2)	0.09	0.74 (0.6, 1.0)	1.1 (0.9, 1.5)	<0.001	0.01
Niacin (mg/day)	6.8 (5.4, 11.4)	9.4 (6.7, 14.7)	0.11	8.9 (5.5, 14.5)	15.5 (12.1, 20.9)	<0.001	<0.001
Vitamin B6 (mg/day)	0.51 (0.4, 0.8)	0.67 (0.5, 1)	0.002	0.69 (0.5, 0.9)	1.0 (0.8, 1.4)	<0.001	<0.001
Folate (μg/day)	95.8 (67.5, 130.8)	115.3 (87.4, 168.6)	0.001	85.8 (59.8, 121.3)	154.0 (127.6, 188.8)	<0.001	0.18
Vitamin B12 (μg/day)	1.5 (0.9, 2.2)	1.7 (0.8, 2.5)	0.69	1.7 (1.2, 3.3)	2.7 (1.8, 3.6)	0.018	0.041
Vitamin C (mg/day)	48.9 (20.3, 70.2)	45.6 (25.6, 61.9)	0.68	38.0 (19.7, 49)	47.8 (36.3, 79.6)	<0.001	0.77
Vitamin E (mg/day)	3.8 (2.9, 5.8)	5.2 (3.7, 7.5)	0.001	4.6 (3.5, 5.7)	7.1 (5.4, 9.4)	<0.001	0.003
**Minerals**							
Calcium (mg/day)	449.2 (313.5, 636.7)	514.8 (349.2, 724.8)	0.68	350.1 (243.5, 518.2)	460.7 (366.7, 667.5)	0.005	0.12
Phosphorus (mg/day)	535.8 (379, 740.5)	629.1 (518.6, 820.6)	0.037	652.8 (426.3, 886.0)	914.2 (732.9, 1096.6)	<0.001	<0.001
Magnesium (mg/day)	146.2 (125.6, 199.3)	192.8 (156.6, 252.7)	0.001	167.3 (129.5, 215.4)	282.1 (227.7, 354.5)	<0.001	<0.001
Iron (mg/day)	8.0 (6.2, 10.0)	9.1 (6.9, 11.2)	0.06	7.1 (5.1, 10.8)	13.8 (11.6, 18)	<0.001	0.01
Copper (μg/day)	480 (440, 780)	650 (490, 890)	0.009	490 (345, 735)	900 (780, 1130)	<0.001	0.03
Sodium (g/day)	1.9 (1.4, 2.7)	2.2 (1.4, 2.9)	0.32	2.0 (1.5, 2.5)	3.0 (2.3, 3.4)	<0.001	0.01
Potassium (g/day)	1.4 (1.2, 1.8)	1.8 (1.5, 2.2)	0.001	1.4 (1.1, 2.1)	2.6 (1.8, 3.0)	<0.001	0.01
Zinc (mg/day)	6.5 (5.2, 7.8)	6.5 (5.3, 7.5)	0.62	6.3 (5.1, 6.7)	6.4 (5.4, 7.4)	0.46	0.63

**Note:** The data are generated by calculating the average daily intake of macro as well as micronutrients from a three-day dietary recall of the study participants at baseline and end of study. Data are presented as median (25th percentile, 75th percentile). The difference within groups (p) at end of study was compared with baseline by Wilcoxon signed-rank test. Interaction *p*-value represents the difference between the groups post intervention. “SF” is saturated fat, “MUFA” and “PUFA” are mono- and polyunsaturated fatty acids, respectively. *p*-value < 0.05 is considered significant.

**Table 4:** Percentage changes in recommended dietary intakes in the two study groups.

Nutrients	RDI (M/F)	GA (N = 75)			Guidance (N = 64)		
		B	ES	*p*	B	ES	*p*
Total Energy	≤RDI_Energy ^1^	40.0	38.7	1.00	39.1	50.0	0.28
**Macronutrients**							
Protein	≤(0.8xBody weight) *	8.0	12.0	0.55	20.3	23.4	0.06
Carbohydrate	≤35% of RDI Energy *	17.3	25.3	0.29	20.3	46.9	0.003
Total Fiber	≥ 25 g/day *	2.7	21.3	0.001	3.1	21.9	0.002
Total Fat	≤25% of RDI Energy *	28.0	33.3	0.56	31.3	39.1	0.38
**Micronutrients**							
Vitamin A	≥ 900/700 μg/day ^₤^	8.0	8.0	1.00	6.3	12.5	0.39
Thiamine	≥ 1.2/1.1 mg/day	1.3	4.0	0.64	4.7	6.3	1.00
Riboflavin	≥ 1.2/1.1 mg/day	15.6	28.1	0.12	10.7	40.0	<0.01
Niacin	≥ 16/14 mg/day ^₤^	20.3	34.4	0.06	14.7	41.3	<0.01
Vitamin B6	≥ 1.3,1.7#/1.3,1.5# mg/day ^₤^	10.9	17.2	0.45	6.7	21.3	0.01
Folate	≥ 400 μg/day *^,₤^	0.0	1.3	-	0.0	1.6	-
Vitamin B12	≥ 2.4 μg/day *	34.4	40.6	0.50	28.0	45.3	0.04
Vitamin C	≥ 90/75 mg/day ^₤^	18.7	21.3	0.83	7.8	21.9	0.02
Vitamin E	≥ 15 mg/day *^,₤^	0.0	0.0	-	0.0	0.0	-
**Minerals**							
Calcium	≥ 1000/1000,1200 ^#^ mg/day ^₤^	6.7	6.7	1.00	4.7	4.7	1.00
Phosphorus	≥ 700 mg/day *^,₤^	38.7	62.7	0.003	40.6	56.3	0.11
Magnesium	≥ 420/320 mg/day ^₤^	4.0	12.0	0.15	4.7	18.8	0.02
Iron	≥ 8/18,8 ^#^ mg/day ^₤^	22.7	40.0	0.02	34.4	54.7	0.03
Copper	≥ 900 μg/day *^,₤^	18.8	34.4	0.08	13.3	37.3	<0.01
Sodium	≤(1.5,1.3^#^,2.3^₤^) * g/day	29.3	12.0	0.007	29.7	21.9	0.38
Potassium	≥ 4.7 * g/day	0.0	0.0	-	0.0	0.0	-
Zinc	≥ 11/8 mg/day ^₤^	14.7	6.7	0.15	4.7	9.4	0.45

**Note**: The data are generated by calculating the percentages of participants following RDI for each macro/micronutrient listed in the table at baseline (B) and end of study (ES). RDA was used only for nutrients where a defined tolerable upper limit (UL) was absent. ^1^ RDI_Energy is calculated by using recommendations by institute of medicine of the national academics and is explained in the methods section. * indicates same RDI for male (M) and female (F); # indicates RDI for age 50 and above; and ₤ indicates UL is considered in the criteria for calculating the percentages. The data are given in percentages of study participants following criteria used in third column. The difference within groups at end of study was compared with baseline by McNemar chi-square test. *p*-value < 0.05 is considered significant.

## Supplementary Materials

The following are available online at https://www.mdpi.com/2072-6643/11/11/2775/s1, Table S1: Other anthropometric and biochemical characteristics of study groups at baseline, Table S2: Other biochemical characteristics of study groups overtime, Table S3: Dietary preferences of study groups’ overtime.
