# Supplementary material for: Improvements in Glycemic, Micronutrient, and Mineral Indices in Arab Adults with Pre-Diabetes Post-Lifestyle Modification Program
Source: Nutrients. 2019 Nov 15;11(11):2775. doi: 10.3390/nu11112775 (PMC6893589; doi:10.3390/nu11112775)
Supplement: Supplementary file 1 [file nutrients-11-02775-s001.pdf]

Article

Improvements in Glycemic and Dietary Nutrient Indices in Arab Adults with Pre-Diabetes post Lifestyle Modification Program

Table S1: Other anthropometric and biochemical characteristics of study groups at baseline:

Parameters	GA (N=75)	Guidance (N=64)	p -Value
Waist (cm)	95.91 ± 12.6	101.3 ± 13.4	**0.02**
Hip circumference (cm)	108.21 ± 9.8	113.27 ± 10.9	**0.01**
Systolic BP (mmHg)	117.35 ± 14.4	123.23 ± 14.1	**0.02**
Diastolic BP (mmHg)	75.08 ± 9.7	78.66 ± 11.3	0.06
Total Cholesterol (mmol/l)	5.11 ± 1.0	5.10 ± 0.9	0.48
LDL-C mmol/l)	3.11 ± 0.9	3.10 ± 0.8	0.97
HDL-C (mmol/l)	1.18 ± 0.4	1.22 ± 0.3	0.46
Triglycerides (mmol/l)	1.47 (1.1,2.03)	1.36 (0.9,1.7)	0.06

Note: LDL-C and HDL_C are low-density and high-density lipoproteins. Data presented as Mean ± SD for continuous normal variables; medians (25th percentile, 75th percentile) for continuous non-normal variables. The difference between groups at baseline was calculated by independent samples t-test and Mann-Whitney U-test for Gaussian and non-Gaussian variables respectively. p<0.05 is taken as significant.

Table S2: Other biochemical characteristics of study groups' overtime.

Parameters		GA (N=75)	Guidance (N=64)
Waist (cm)	Baseline	95.91 ± 12.6	101.30 ± 13.4
	6-months	97.84 ± 12.2	100.58 ± 13.4
Hip circumference (cm)	Baseline	108.21 ± 9.8	113.27 ± 10.9
	6-months	109.57 ± 10.0	110.59 ± 11.0**
Systolic BP (mmHg)	Baseline	117.35 ± 14.4	123.23 ± 14.1
	6-months	115.72 ± 10.7	116.95 ± 12.2**
Diastolic BP (mmHg)	Baseline	75.08 ± 9.7	78.66 ± 11.3
	6-months	74.61 ± 7.8	75.39 ± 9.6*
Total Cholesterol (mmol/l)	Baseline	5.11 ± 1.0	5.00 ± 0.9
	6-months	5.07 ± 1.1	4.78 ± 1.0*
HDL-C (mmol/l)	Baseline	1.18 ± 0.4	1.22 ± 0.3
	6-months	1.09 ± 0.4	1.17 ± 0.4
LDL-C mmol/l)	Baseline	3.11 ± 0.9	3.10 ± 0.8
	6-months	3.25 ± 1.0	2.96 ± 0.9
Triglycerides (mmol/l)	Baseline	1.47 (1.1,2.2)	1.36(1.0,1.7)
	6-months	1.39 (1.0,1.8)	1.26(0.9,1.6)

Note: LDL-C and HDL_C are low-density and high-density lipoproteins. Data presented as Mean ± SD for continuous normal variables; medians (25th percentile, 75th percentile) for continuous non-normal variables. The difference within groups compared with baseline was calculated by repeated measures t-test and Wilcoxon signed-rank test for Gaussian and non-Gaussian variables respectively. **depicts p-value<0.01 and * depicts p-value<0.05.

Table S3: Dietary preferences of study groups' overtime.

		GA (N=75)		Guidance (N=64)	
		Median (Q1, Q3)	p	Median (Q1, Q3)	p
Full Fat Dietary Products/week	Baseline	2.50 (1.6 , 4.0)	0.272	2.56 (1.5 , 4.6)	**0.04**
	6-months	3.00 (2.0 , 4.0)		2.00 (1.6 , 3.0)	
Low Fat Dietary Products/week	Baseline	1.40 (1.1 , 3.1)	0.222	1.40 (1.1 , 2.2)	**0.003**
	6-months	1.45 (1.1 , 2.4)		1.72 (1.3 , 2.8)	
Red Meat/week	Baseline	1.50 (0.8 , 3.5)	0.687	1.50 (0.8 , 3.5)	0.076
	6-months	1.50 (1.5 , 3.5)		1.50 (0.8 , 1.5)	
White Meat/week	Baseline	3.00 (1.0 , 7.0)	**0.010**	3.00 (3.0 , 3.0)	**0.016**
	6-months	3.00 (3.0 , 7.0)		3.00 (3.0 , 7.0)	
Fish Meat/week	Baseline	0.25 (0.1 , 1.0)	0.570	0.25 (0.1 , 0.3)	**0.038**
	6-months	0.25 (0.1 , 1.0)		0.25 (0.2 , 1.0)	
Pulses, Legumes or Beans/week	Baseline	1.50 (1.5 , 3.5)	0.650	1.50 (0.8 , 3.5)	0.815
	6-months	1.50 (1.5 , 3.5)		1.50 (1.5 , 2.3)	
Fruits and/or Vegetables/week	Baseline	3.00 (2.0 , 5.0)	0.114	2.00 (1.0 , 3.0)	0.493
	6-months	3.00 (1.6 , 5.0)		2.00 (1.0 , 4.0)	
White Bread/week	Baseline	6.00 (3.0 , 6.0)	0.523	6.00 (3.0 , 6.0)	**<0.01**
	6-months	6.00 (3.0 , 6.0)		4.00 (3.0 , 6.0)	
Whole meal Bread/week	Baseline	1.50 (0.1 , 3.5)	0.102	1.25 (0.5 , 3.0)	**<0.01**
	6-months	0.56 (0.1 , 3.5)		1.50 (0.2 , 3.5)	
Soft drinks and/or Fruit Juices/week	Baseline	3.00 (1.5 , 4.5)	0.301	2.25 (1.4 , 4.1)	0.166
	6-months	3.00 (2.0 , 4.5)		2.25 (1.4 , 3.0)	

Note: The data represents the dietary preferences of the participants in the two study groups at baseline and after intervention calculated as frequency/week. Data is presented as median (25th percentile, 75th percentile). The difference within groups at end of study was compared with baseline by Wilcoxon signed-rank test. P-value<0.05 is considered significant.

Table S3 shows the changes in dietary preferences of the two study groups at baseline and end of study. Participants in the guidance group reduced their consumption of full fat dietary products from median 2.56 times to 2.00 times per week ($p<0.05$), while preferred to consume more of low fat dietary products instead from 1.40 times to 1.70 times per week ($p<0.01$). The consumption of red meat in this group was 1.50 times/week and didn't change overtime, however, white meat (chicken etc) and fish meat consumption increased modestly at end of study compared to baseline. Similarly, in this group, the consumption of white bread decreased from 6 times/week to 4 times/week on an average ($p<0.01$) while it got replaced with whole meal bread products from 1.25 to 1.50 times/week ($p<0.01$).
